# Cost of specialized addiction treatment of clients with fetal alcohol spectrum disorder in Canada

**DOI:** 10.1186/1471-2458-13-570

**Published:** 2013-06-11

**Authors:** Svetlana Popova, Shannon Lange, Larry Burd, Karen Urbanoski, Jürgen Rehm

**Affiliations:** 1 1Social and Epidemiological Research Department, Centre for Addiction and Mental Health, 33 Russell St., Toronto, ON M5S 2S1, Canada; 2 2Dalla Lana School of Public Health, University of Toronto, 155 College St, Toronto, ON M5T 3M7, Canada; 3 3Factor-Inwentash Faculty of Social Work, University of Toronto, 246 Bloor St W, Toronto, ON M5S 1V4, Canada; 4 4Institute of Medicine, University of Toronto, 1 King's College Circle, Toronto, ON M5S 1A8, Canada; 5 5Department of Pediatrics, University of North Dakota School of Medicine, 501 North Columbia Rd, Grand Forks, ND 58203, USA; 6 6Epidemiological Research Unit, Klinische Psychologie and Psychotherapie, Technische Universität Dresden, Chemnitzer Str 46, Dresden D-01187, Germany

**Keywords:** Fetal alcohol syndrome, Fetal alcohol spectrum disorder, Addiction, Specialized treatment, Utilization, Cost, Canada

## Abstract

**Background:**

Individuals with Fetal Alcohol Spectrum Disorder (FASD) constitute a special population that may be at particularly high risk for substance use. The purpose of the current study was to estimate the utilization of specialized addiction treatment services (SATS) and the associated cost, as a part of the total cost of health care associated with FASD in Canada.

**Methods:**

The current study was a modeling study. Data on SATS by lifetime mental disorder status were obtained from the Drug and Alcohol Treatment Information System (DATIS) in Ontario, Canada for 2010/11. The number of clients with FASD who received SATS in Ontario in 2010/11 was estimated, assuming that approximately 37% (confidence interval: 21.6%-54.5%) of individuals with FASD abuse or are addicted to alcohol and/or drugs and that their utilization rate of SATS is the same as those for people with a lifetime mental disorder. The data from DATIS was then extrapolated to the total Canadian population.

**Results:**

The cost of SATS for clients with FASD in Canada in 2010/11 ranged from $1.65 million Canadian dollars (CND) to $3.59 million CND, based on 5,526 outpatient visits and 9,529 resident days. When the sensitivity analysis was performed the cost of SATS ranged from $979 thousand CND to $5.34 million CND.

**Conclusions:**

Special attention must be paid to at-risk groups of individuals such as those with FASD, in order to reduce the likelihood of the development of co-morbid substance abuse problems, and thus, reducing the overall burden on Canadian society.

## Background

This study is a part of a large economic study on the estimation of the burden and cost associated with Fetal Alcohol Spectrum Disorder (FASD) in Canada [1,2]. FASD is a non-diagnostic term that encompasses four alcohol-related clinical diagnoses, including: Fetal Alcohol Syndrome (FAS), Partial FAS, Alcohol-Related Neurodevelopmental Disorder, and Alcohol-Related Birth Defects.

Individuals with FASD have an increased vulnerability to certain risk factors, which put them at a higher risk for substance use/abuse problems, as a result of the damage caused to their central nervous system due to their exposure to alcohol prenatally. This damage manifests as developmental delays, cognitive impairments, mental disorders, high rates of incarceration and an increased rate of substance abuse [3-5].

The impairments expressed by individuals with FASD typically lead to high-risk behaviours, such as alcohol/drug abuse or an increased likelihood of being in high-risk situations (increasing the chance of being exposed to alcohol and/or drugs). Currently, there are not many epidemiological studies reporting on the prevalence of substance use/abuse among individuals with FASD. However, the literature does indicate that a disproportionate number of individuals with FASD will have problematic substance use issues at some point in their lives [6]. For instance, Famy et al. [7] reported a prevalence of 55% for alcohol/drug dependence among their sample of individuals with FAS. Additionally, Clark and colleagues [8] and Streissguth and colleagues [9] reported that 22% and 35% of their respective cohorts of individuals with FASD had problems with alcohol/drugs. Lastly, in a study conducted by Grant and colleagues [10], about 68% of women with FASD had reported abusing alcohol and 79% had used illegal drugs in the 10 months prior to being admitted into a community program.

There are a few potential explanations for the high prevalence of substance use among individuals with FASD: 1) a biological vulnerability to substance use may exist; 2) individuals with FASD may use substances to self-medicate; and 3) individuals with FASD tend to have difficulties with impulse control, making them susceptible to developing a substance use disorder [11]. Regardless of the reason for such high rates of co-occurring substance use problems and FASD, this population places a greater demand on treatment service providers, given that they suffer from multiple co-morbid mental disorders [8,12,13].

Prenatal alcohol use exposes three generations to the harmful effects of alcohol (the mother, the fetus, and the germ line of the fetus). Increased rates of substance abuse increase the risk for additional familial cases of FASD (multiple affected siblings), and increase the risk of generational FASD [3,14]. Therefore, it is also important to address the issue of women of childbearing age with FASD so that the generational effects of FASD can be halted.

In Canada, as is common elsewhere, specialized addiction treatment services provide a continuum of care, from assistance with symptoms of withdrawal through to active treatment and continuing care [15]. They vary, however, in their capacity for assisting clients with co-occurring disorders [15,16], which would include complications arising from FASD among others. Prior research suggests that people with co-morbid substance use and mental disorders have a more complicated trajectory of service use, including higher rates of relapse and readmission to addiction treatment [17-22]. Given the high-risk nature of FASD for multiple health and social comorbidities, it is of interest to understand their representation in the specialized addiction treatment system.

The purpose of this study was to estimate the use of specialized addiction treatment services and the associated cost, as a part of the total cost of health care associated with FASD in Canada.

## Methods

Since there is great uncertainty regarding the true prevalence of people with FASD in the general population, and the prevalence of people with FASD who abuse or are addicted to substances, specifically, in combination with the lack of FASD-specific data pertaining to specialized addiction treatment services in Canada, several important assumptions were used and sensitivity analyses were conducted, as described step-by-step below.

### Source of data

Data were obtained from the Drug and Alcohol Treatment Information System (DATIS; http://www.datis.ca), which monitors the use of specialized addiction treatment services by people in Ontario, Canada. Started in 1992, DATIS collects data on the numbers and types of clients entering publicly funded specialized addiction treatment across the province. This system includes approximately 200 treatment programs, administered by 170 agencies [23,24]. The types of services provided by each agency can vary; some agencies provide a specific type of service (e.g., assessment and referral, withdrawal management, individual counseling), while others provide a comprehensive mix of services. Regardless of the type of service, all services are delivered free-of-charge to Ontario residents (i.e., they are covered by the province’s universal health insurance plan). Agency-level participation has been over 95% since 2000.

The DATIS database is structured by admissions to treatment programs, such that a new admission is triggered when a client enters a new treatment program or transitions between two different types of services. Since 2002, data entry has been supported by a web-based user platform accessible by all frontline clinicians working in the designated programs across the province. Data entry fields correspond to 66 data elements that are mandatory for all admissions. Sociodemographic characteristics, information on substance use, and other treatment-related factors (e.g., referral source, current and past diagnoses of mental disorders, and treatment mandates) are entered by the service provider at admission, typically following the first face-to-face encounter with the client. Unique identifiers for individual clients and agencies are generated automatically by the software, as is a variable documenting the type of treatment program or service (i.e., outpatient, residential or residential withdrawal management). Details on service use, including the number of outpatient visits and days or residential care, are entered by service providers at discharge. Data are stored on a central server located at the Centre for Addiction and Mental Health in Toronto, Canada.

### Study population

All admissions corresponding to services received during the 2010/11 fiscal year (April 1, 2010 to March 31, 2011; N=91,333) were extracted for analysis. This included all admissions occurring during the fiscal year, as well as those where treatment had started prior to April 1, 2010, but that had continued into the study period. As noted above, an admission corresponds to a particular type of service, with movement between types of services (e.g., from a residential service to an outpatient program) counted as separate admissions. Multiple admissions per individual client were included. This was done to ensure that the most complete estimates of the volume and costs of services delivered during the study period were obtained. Therefore, it is important to note that each admission does not necessarily represent a separate individual, as one individual can have multiple admissions.

### Data elements

In order to determine whether or not a client has had a “lifetime mental disorder” (i.e., a diagnosed mental disorder at any point within their lifetime; response: yes versus no), the question “Have you ever been diagnosed by a qualified mental health professional with a mental disorder within the last 12 months or within your lifetime?” was asked to each client at the beginning of treatment.

Age groups, divided into 5-year intervals from 14 years of age and younger to 70 years of age and older, were generated from the clients’ date of birth. All variables (lifetime mental disorder, date of birth, and sex) were self-reported by the clients during their initial clinical encounter.

Service type (i.e., outpatient, residential treatment, or residential withdrawal management, automatically generated in DATIS) was abstracted, as were the number of visits for outpatient treatment and days in residential treatment and residential withdrawal management. The numbers of visits and days of care, entered by service providers based on client charts, provide estimates of the volume of services received. For admissions that began prior to or ended after the 2010/11 fiscal year, only those visits/days that occurred within the study period were counted. To comply with Personal Health Information Protection Act (2004), cells with values less than 6 were redacted and replaced with "<6".

### Estimation of the prevalence of clients with FASD in specialized addiction treatment and their utilization of these services

#### Number of people with FASD in Canada

The prevalence of FAS and FASD are currently unknown in Canada. However, the most commonly cited rough estimates are 1 per 1,000 for FAS [25], and 9 per 1,000 for FASD [26]. Using data on the general population, by age group and sex, of Canada in 2010 [27], and assuming a prevalence of 9 per 1,000 for FASD [26], the number of people with FASD in Canada was estimated.

### Number of people with FASD who abuse or are addicted to alcohol and/or drugs

In order to estimate the number of clients with FASD who had received specialized addiction treatment services in Canada in 2010/11, the prevalence of individuals with FASD who abuse or are addicted to alcohol and/or drugs was calculated based on available epidemiological studies [7-10] using the meta-analysis technique described below.

### Meta-analysis

Prevalence data from the epidemiological studies concerning alcohol/drug abuse/use/dependence among those with FASD were transformed into log-odds for the meta-analysis [28]. Log-odds estimates were weighted by the inverse variance of the log-odds transformed prevalence. Heterogeneity between studies was assessed using the Cochrane Q-test and the I^2^ statistic [29,30]. The prevalence estimates were pooled using the Mantel-Haenszel method, using a random-effects model [31].

Publication bias was tested by: 1) visually inspecting a funnel plot for skewed distribution, 2) using a ranked correlation test [32], and 3) employing a weighted regression test [33]. Publication bias was then adjusted for using the trim and fill method [34].

### Number of people with FASD who utilized specialized addiction treatment services and their rate of utilization in 2010/11

In order to calculate the number of people with FASD who utilized specialized addiction treatment services in 2010/11, it was assumed that the rate of specialized addiction treatment services among this population (individuals with FASD) was the same as the rate among individuals with a lifetime mental disorder. This assumption is based on a very high prevalence of co-morbid mental illness reported among individuals with FASD [7,9,35,36]. Based on the authors’ comprehensive literature review (Popova et al., unpublished), the weighted mean for mental retardation (International Classification of Diseases, version 10 [ICD-10] category: F70-F79) among individuals with FASD is 48% (95% confidential interval [CI]: 44.4%-51.4%) and for disorders of psychological development (ICD-10 category: F80-F89) is 37% (95% CI: 35.9%-39.0%).

In order to estimate the rate of utilization of specialized addiction treatment services for individuals with a lifetime mental disorder, the number of specialized addiction treatment services admissions for individuals with a lifetime mental disorder was divided by the total number of individuals with a mental illness in Ontario (reported by the Ministry of Health and Long-Term Care [36] and Health Canada [37]).

In turn, in order to estimate the total number of admissions, and visits/days, by treatment type, among clients with FASD for all of Canada, the distribution for each treatment type among clients who received specialized addiction treatment services in the province of Ontario was used and extrapolated to the total Canadian population. This approach is justifiable given that Ontario represents about 39% of the total population of Canada.

### Estimation of costs

The cost for specialized outpatient treatment ranged from $60 to $109 per service and for residential treatment ranged from $138 to $314 per resident day in Canada in 2010/11 (Martin et al., in progress). These unit costs are estimated based on the costs reported by five Local Health Integration Networks (LHINs) across Ontario. The overall estimates are inclusive of the cost of supervision, facility costs, salaries, and other sundry expenses. The ranges reported account for the differences in costs incurred due to the number of spots/beds available (capacity), whether the treatment is hospital based or community based, the intensity of activities provided, and staff professionalism (which affects both their salaries and the scope of the staff complement).

The corresponding costs - for outpatient treatment: $60 and $109 per service as the lower and upper estimates, respectively; and for residential treatment: $138 and $314 per resident day as the lower and upper estimates, respectively - were applied to substance-attributable specialized outpatient visits and residential days, in order to obtain the total costs of such services for clients with FASD.

All cost figures are presented in Canadian dollars.

### Sensitivity analysis

Due to a very limited number of existing epidemiological studies, there is great uncertainty regarding the prevalence of individuals with FASD who abuse or are addicted to alcohol and/or drugs. As described above, the weighted mean of 37% (CI: 21.6%-54.5%), which was calculated based on the available epidemiological studies [7-10] was used in the main analysis. In addition, two separate analyses were performed assuming that 22% (as the lower estimate) and 55% (as the upper estimate; both are based on the estimated CI) of individuals with FASD abuse or are addicted to alcohol and/or drugs.

## Results

### Calculation of the rate of specialized addiction treatment service among individuals with a lifetime mental disorder

Given that 20% of the population, age 12 and over, of Ontario [37] reported a mental health diagnosis such as mood disorders, anxiety disorders, and/or schizophrenia in 2005 and based on the population size of Ontario (13,227,791 [27]), the total number of individuals who suffer with a mental illness in Ontario was calculated to be 2,645,558. The rate of 20% has also been reported for all of Canada [38].

In order to estimate the rate of utilization of specialized addiction treatment services for individuals with a lifetime mental disorder, the number of specialized addiction treatment services admissions for clients with a lifetime mental disorder (N=37,164; obtained from DATIS for 2010/11) was divided by the total number of individuals with a mental illness in Ontario (2,645,558), resulting in the rate of 1.4%. The rate of utilization of specialized addiction treatment services for individuals without a lifetime mental disorder was estimated to be almost three times lower (i.e., 0.5%).

### Estimation of the number of clients with FASD who received specialized addiction treatment services in Ontario and Canada in 2010/11

Using data on the general population of Ontario in 2010 (13,227,791 [27]) and assuming a prevalence of 9 per 1,000 for FASD [26], it was estimated that there were 119,050 individuals with FASD in Ontario in 2010/11.

Results of the meta-analysis revealed that approximately 37% of individuals with FASD abuse or are addicted to alcohol and/or drugs. Applying 37% to 119,050 individuals with FASD, results in 44,049 of individuals with FASD who abuse or are addicted to alcohol and/or drugs in Ontario in 2010/11. Assuming that the utilization rate of specialized addiction treatment services among individuals with FASD is the same as those for people with a lifetime mental disorder (1.4%; see above), the number of admissions for clients with FASD who received specialized addiction treatment services in Ontario in 2010/11 was estimated to be 617.

Similarly, using data on the general population of Canada in 2010 (34,126,181 [27]) and an assumed prevalence of 9 per 1,000 for FASD [26], it was estimated that there were 307,136 individuals with FASD in Canada in 2010/11.

Again, under the assumption that approximately 37% (113,640) of individuals with FASD abuse or are addicted to alcohol and/or drugs and that the utilization rate of specialized addiction treatment services among individuals with FASD is the same as those for people with a lifetime mental disorder (1.4%; see above), the number of admissions for clients with FASD who received specialized addiction treatment services in Canada in 2010/11 was estimated to be 1,591.

### Service utilization and estimation of the cost associated with addiction treatment services for clients with FASD

Based on the service rates obtained from DATIS, and the assumption that individuals with FASD have the same utilization rates as those of individuals with a lifetime mental disorder, it was estimated that there were 1,591 admissions in total in Canada (546 for outpatient, 189 for residential, and 856 for withdrawal management) among clients with FASD. These admissions resulted in 5,526 outpatient visits, and 9,529 residential days; the associated cost was estimated to range from $1.65 million to $3.59 million.

The number of admissions, visits/days in outpatient treatment, residential treatment, and residential withdrawal management services and costs among clients with FASD by age groups and sex in Ontario and Canada in 2010/11 are presented in Tables 1, 2, and 3, respectively.

**Table 1:** Estimated number of admissions, visits and cost of outpatient treatment services among clients with FASD by age groups and sex in Ontario and Canada in 2010/11

**Gender**	**Age group**														**Cost**	
	**14 and under**	**15-19**	**20-24**	**25-29**	**30-34**	**35-39**	**40-44**	**45-49**	**50-54**	**55-59**	**60-64**	**65-69**	**70 and over**	**All ages**	**Lower estimate**	**Upper estimate**
**Clients with FASD in Ontario**																
**Number of admissions**																
Male	<6	15	13	11	12	11	13	13	10	7	<6	<6	<6	112		
Female	<6	10	11	13	13	12	11	12	8	<6	<6	<6	<6	99		
***Total***	***<6***	***25***	***24***	***24***	***25***	***23***	***24***	***25***	***18***	***11***	***6***	***<6***	***<6***	***212***		
**Number of visits**																
Male	17	98	91	93	124	124	143	137	111	90	29	14	7	1,078	$64,670	$117,484
Female	23	82	106	143	127	121	127	147	79	56	31	15	9	1,065	$63,914	$116,111
***Total***	***40***	***180***	***197***	***237***	***250***	***245***	***270***	***283***	***190***	***146***	***60***	***29***	***16***	***2,143***	***$128,585***	***$233,595***
**Clients with FASD in Canada**																
**Number of admissions**																
Male	6	38	32	29	31	29	34	33	26	18	8	<6	<6	290		
Female	<6	26	30	34	32	30	28	31	20	12	6	<6	<6	256		
***Total***	***10***	***63***	***62***	***63***	***64***	***59***	***61***	***64***	***46***	***29***	***14***	***6***	***<6***	***546***		
**Number of visits**																
Male	43	251	236	241	319	320	369	353	286	232	75	37	18	2,779	$166,759	$302,946
Female	59	212	272	370	326	312	327	378	204	145	81	38	22	2,747	$164,810	$299,405
***Total***	***102***	***463***	***508***	***610***	***645***	***632***	***696***	***731***	***490***	***377***	***156***	***74***	***40***	***5,526***	***$331,569***	***$602,350***

FASD: Fetal Alcohol Spectrum Disorder.

**Note.** Cells with values less than 6 were redacted and replaced with "<6".

**Table 2:** Estimated number of admissions, resident days and cost of residential treatment services among clients with FASD age groups and sex in Ontario and Canada in 2010/11

**Gender**	**Age group**														**Cost**	
	**14 and under**	**15-19**	**20-24**	**25-29**	**30-34**	**35-39**	**40-44**	**45-49**	**50-54**	**55-59**	**60-64**	**65-69**	**70 and over**	**All ages**	**Lower estimate**	**Upper estimate**
**Clients with FASD in Ontario**																
**Number of admissions**																
Male	<6	<6	<6	<6	6	7	6	6	5	<6	<6	<6	<6	44		
Female	<6	<6	<6	<6	<6	<6	<6	<6	<6	<6	<6	<6	<6	29		
***Total***	***<6***	***<6***	***8***	***11***	***10***	***10***	***9***	***10***	***7***	***<6***	***<6***	***<6***	***<6***	***73***		
**Number of days**																
Male	<6	99	158	200	234	264	235	292	223	93	41	9	7	1,855	$256,056	$582,621
Female	<6	84	104	174	143	116	114	102	90	49	10	<6	<6	989	$136,472	$310,524
***Total***	***7***	***183***	***262***	***374***	***376***	***381***	***349***	***394***	***313***	***142***	***51***	***12***	***7***	***2,844***	***$392,529***	***$893,145***
**Clients with FASD in Canada**																
**Number of admissions**																
Male	<6	6	11	14	15	17	14	16	13	6	<6	<6	<6	115		
Female	<6	<6	10	13	10	9	9	9	<6	<6	<6	<6	<6	74		
***Total***	***<6***	***10***	***21***	***27***	***25***	***25***	***23***	***25***	***18***	***9***	***<6***	***<6***	***<6***	***189***		
**Number of days**																
Male	13	254	407	515	603	682	607	753	576	241	105	24	18	4,785	$660,269	$1,502,351
Female	<6	217	267	450	368	300	294	263	231	126	27	6	<6	2,550	$351,908	$800,718
***Total***	***18***	***471***	***675***	***965***	***971***	***982***	***900***	***1,016***	***807***	***367***	***131***	***30***	***19***	***7,335***	***$1,012,177***	***$2,303,069***

FASD: Fetal Alcohol Spectrum Disorder.

**Note.** Cells with values less than 6 were redacted and replaced with "<6".

**Table 3:** Estimated number of admissions, resident days and cost of residential withdrawal management services among clients with FASD age groups and sex in Ontario and Canada in 2010/11

**Gender**	**Age group**														**Cost**	
	**14 and under**	**15-19**	**20-24**	**25-29**	**30-34**	**35-39**	**40-44**	**45-49**	**50-54**	**55-59**	**60-64**	**65-69**	**70 and over**	**All ages**	**Lower estimate**	**Upper estimate**
**Clients with FASD in Ontario**																
**Number of admissions**																
Male	<6	7	19	29	31	28	31	30	24	14	<6	<6	<6	219		
Female	<6	<6	15	18	16	13	14	13	10	<6	<6	<6	<6	111		
***Total***	***<6***	***12***	***34***	***47***	***48***	***42***	***45***	***43***	***33***	***19***	***6***	***<6***	***<6***	***332***		
**Number of days**																
Male	<6	20	41	61	70	83	90	88	75	30	23	<6	<6	587	$80,941	$184,169
Female	<6	10	35	36	40	28	36	28	29	13	<6	<6	<6	264	$36,455	$82,948
***Total***	***<6***	***30***	***76***	***97***	***110***	***111***	***126***	***117***	***104***	***43***	***28***	***<6***	***<6***	***851***	***$117,438***	***$267,213***
**Clients with FASD in Canada**																
**Number of admissions**																
Male	<6	18	49	74	81	73	81	77	61	37	12	<6	<6	566		
Female	<6	14	37	47	42	35	36	32	25	12	<6	<6	<6	287		
***Total***	***<6***	***32***	***87***	***121***	***123***	***107***	***117***	***110***	***86***	***49***	***16***	***<6***	***<6***	***856***		
**Number of days**																
Male	<6	51	105	157	180	214	232	228	194	78	60	8	7	1,512	$208,714	$474,901
Female	<6	26	91	94	104	73	94	73	74	33	12	<6	<6	681	$94,003	$213,891
***Total***	***<6***	***77***	***196***	***251***	***285***	***286***	***325***	***301***	***268***	***111***	***72***	***12***	***9***	***2,194***	***$302,825***	***$689,037***

FASD: Fetal Alcohol Spectrum Disorder.

**Note.** Cells with values less than 6 were redacted and replaced with "<6".

**Figure 1:**
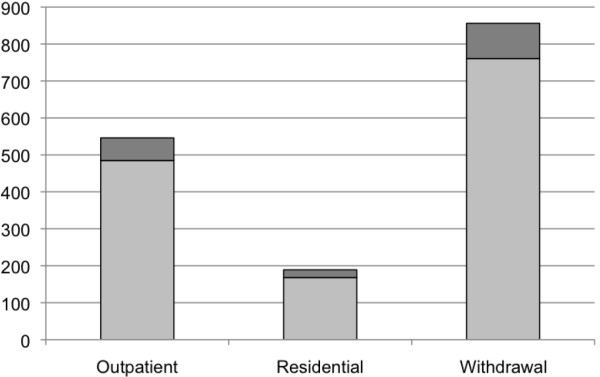
**Estimated number of admissions among clients with FASD (including FAS**^**a**^**) in Canada 2010/11. **^a^ Estimated based on a prevalence of 1 per 1,000 [21].

**Figure 2:**
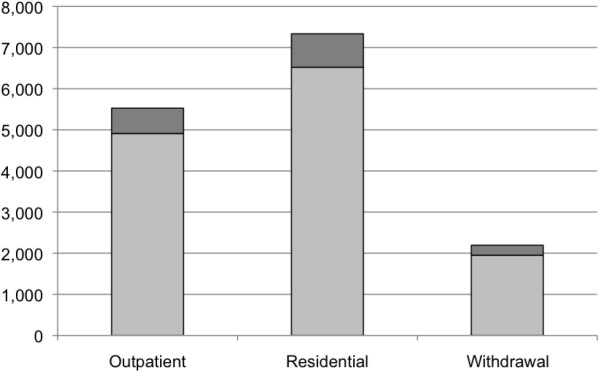
**Estimated number of visits/inpatient days among clients with FASD (including FAS**^**a**^**) in Canada 2010/11. **^a^ Estimated based on a prevalence of 1 per 1,000 [21].

**Figure 3:**
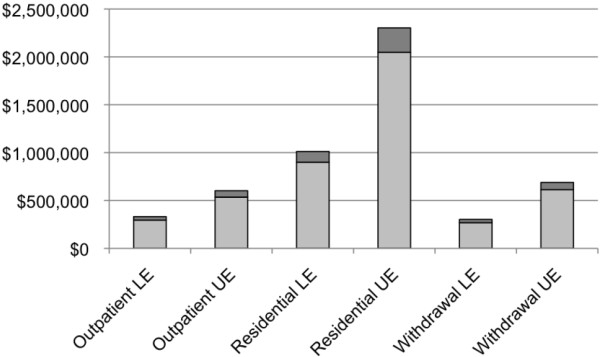
**The overall estimated costs associated with specialized addiction treatment among clients with FASD (including FAS**^**a**^**) in Canada 2010/11.** LE: Lower Estimate. UE: Upper Estimate. ^a^ Estimated based on a prevalence of 1 per 1,000 [21].

The total number of admissions, visits/days and costs of specialized addiction treatment services among clients with FASD for Ontario and Canada, as a whole, in 2010/11 are presented in Table 4 and Figures 1, 2 and 3 (FAS is figuratively presented separately).

**Table 4:** Estimated number of admissions, outpatient visits, resident days and costs of specialized addiction treatment services among clients with FASD in Ontario, and Canada in 2010/11

	**Number of admissions, outpatient visits and resident days; associated cost (lower & upper estimate)**	**Total population (2010)**^**a**^	**Number of clients with FASD (based on a prevalence of 9 per 1,000)**	**Types of specialized addiction treatment services**			
				**Outpatient**	**Residential**	**Withdrawal**	**Overall**
**Ontario**		**13,227,791**	**119,050**				
	Number of admissions			212	73	332	**617**
	Number of visits			2,143			**2,143**
	Number of days				2,844	851	**3,695**
	Cost (Lower estimate)			$128,585	$392,529	$117,438	**$638,551**
	Cost (Upper estimate)			$233,595	$893,145	$267,213	**$1,393,953**
**Canada (All provinces/territories)**		**34,126,181**	**307,136**				
	Number of admissions			546	189	856	**1,591**
	Number of visits			5,526			**5,526**
	Number of days				7,335	2,194	**9,529**
	Cost (Lower estimate)			$331,569	$1,012,177	$302,825	**$1,646,571**
	Cost (Upper estimate)			$602,350	$2,303,069	$689,037	**$3,594,456**
**1) Sensitivity analysis, assuming 22% (lower estimate) of individuals with FASD suffer from addiction**		**34,126,181**	**307,136**				
	Number of admissions			325	112	509	**946**
	Number of visits			3,286			**3,286**
	Number of days				4,361	1,305	**5,666**
	Cost (Lower estimate)			$197,144	$601,866	$180,029	**$979,037**
	Cost (Upper estimate)			$358,145	$1,369,464	$409,626	**$2,137,235**
**2) Sensitivity analysis, assuming 55% (upper estimate) of individuals with FASD suffer from addiction**		**34,126,181**	**307,136**				
	Number of admissions			812	281	1,272	**2,365**
	Number of visits			8,214			**8,214**
	Number of days				10,903	3,261	**14,165**
	Cost (Lower estimate)			$492,859	$1,504,666	$450,066	**$2,447,592**
	Cost (Upper estimate)			$895,361	$3,423,661	$1,024,064	**$5,343,086**

^a^ Source: Statistics Canada [23].

FASD: Fetal Alcohol Spectrum Disorder.

**Note.** Columns may not add up due to rounding errors.

Please note that the number of admissions, visits/days and costs of specialized addiction treatment services by province/territory among clients with FASD in 2010/11 are available from the authors upon request.

On average, in Canada, there were per admission approximately 10 visits for outpatient treatment; 39 days for residential treatment; and 3 days for residential withdrawal management services.

**Figure 4:**
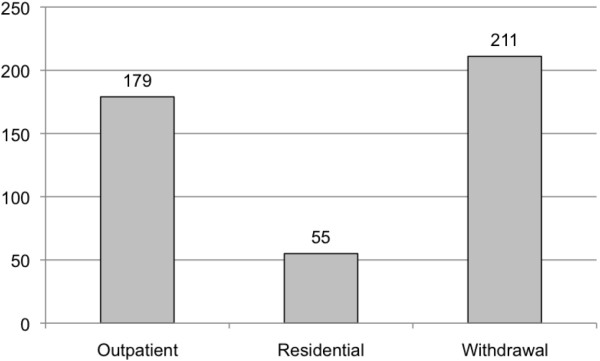
Estimated number of admissions among female clients of childbearing age with FASD in Canada 2010/11.

**Figure 5:**
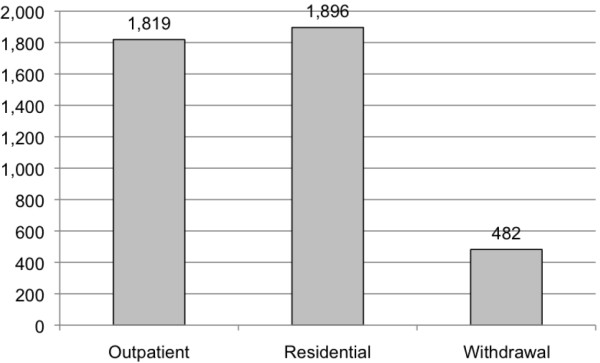
Estimated number of visits/inpatient days among female clients of childbearing age with FASD in Canada 2010/11.

Further, it was estimated that in Canada in 2010/11 there were approximately 445 specialized addiction treatment services admissions of women of childbearing age (15–44) with FASD (Figure 4). These admissions resulted in 1,819 outpatient visits and 2,378 residential days (1,896 days in residential treatment, and 482 days in residential withdrawal treatment; Figure 5).

### Sensitivity analysis

Assuming that 22% (as the lower estimate) of individuals with FASD abuse or are addicted to alcohol and/or drugs, it was estimated that there were 946 admissions in total (325 for outpatient, 112 for residential, and 509 for withdrawal management) in Canada in 2010/11 - resulting in 3,286 outpatient visits and 5,666 resident days. The associated cost was estimated to range from $979 thousand to $2.14 million (Table 4).

Assuming that 55% (as the upper estimate) of individuals with FASD abuse or are addicted to alcohol and/or drugs, it was estimated that there were 2,365 admissions in total (812 for outpatient, 281 for residential, and 1,272 for withdrawal management) in Canada in 2010/11. These admissions resulted in 8,214 outpatient visits and 14,165 resident days. The associated cost was estimated to range from $2.45 million to $5.34 million (see Table 4).

## Discussion

It is clear from the current estimated costs associated with specialized addiction treatment services for clients with FASD in Canada that substance abuse problems among this specific population place an additional economic burden on Canadian society. It is important to note that the cost of specialized addiction treatment services, estimated in the current study, is only a part of the total cost of health care associated with FASD [1,2,39,40].

As the results indicate, individuals with a co-morbid mental disorder represent a large portion of those utilizing specialized addiction treatment services in Canada – about 40%. This has also been supported by previous research [41-43]. Within this group are those with FASD, which represent a population that is high-risk for concurrent mental health disorders [7,12,13,44,45]. Research shows that such high-risk populations with co-morbid mental disorders have higher rates of relapse and readmission to treatment, and thus, have a more complicated trajectory of service use and place a greater demand on service providers [17-22].

Individuals with FASD who abuse substances constitute a special population for service providers for multiple reasons. First, all individuals with FASD have an affected family member and many will have multiple affected family members [14,46,47]. Second, nearly all individuals with FASD have neurocognitive impairments that will affect their response to substance abuse treatment [12]. Third, many, if not most individuals with FASD entering substance abuse treatment will not be recognized as having FASD. This is especially true in the correctional system [48,49]. Fourth, very few substance abuse treatment programs have the needed adaptations for clients with FASD. This gap in services may increase their risk of treatment failure and/or relapse. Fifth, treatment strategies of women with FASD should be different from those for men [50]. An essential goal for women with FASD must be to increase their knowledge of the effects of prenatal alcohol exposure and a treatment goal must be no use of alcohol after their child bearing years. This is crucial since no known level of alcohol use during pregnancy has been deemed to be safe. While some interventions focus on birth control, it is also important to acknowledge that this intervention is not 100% effective, and that some women will not adhere to treatment protocols. In the current study, it was estimated that each month women of childbearing age are responsible for about 18 admissions in withdrawal management programs in Canada.

There are several limitations of the current study that are worth mentioning. First, the prevalence of FASD is unknown in Canada; therefore, for the purpose of this analysis, the most commonly cited rough estimate of the prevalence of FASD (9 per 1,000 [26]) was used, which may not be accurate. Second, the utilization rate of specialized addiction treatment services by individuals with FASD is unknown in Canada; therefore, it was assumed that their utilization rate is the same as those for people with a lifetime mental disorder. Third, the number of individuals with a lifetime mental disorder may be underestimated in the current study due to at least two reasons: 1) self-reported data is a subject to recall bias; and 2) by asking clients to self-report mental diagnoses, those who have not previously sought services or have not received a diagnosis were excluded. As such, using the assumption that the utilization rate of specialized addiction treatment services among individuals with FASD is the same as that of people with a lifetime mental illness, it is likely that the utilization rate of individuals with FASD is also underestimated in the current study.

The data presented here on the number of women of childbearing age (15 to 44 years) with FASD in substance abuse treatment (approximately 450 admissions) highlights the increased risk for additional FASD cases among the future children of these women. Based on these data, there is an evident need for additional training for programs serving this population. System-wide screening strategies and targeted interventions for women of childbearing age with FASD also need to be developed. These recommendations should be primary prevention priorities for substance abuse prevention programs.

## Conclusions

Special attention must be paid to at-risk groups of individuals – such as those with FASD, in order to reduce the likelihood of the development of secondary disabilities (in this case, substance abuse problems). Reducing the occurrence of substance abuse problems will alleviate a portion of the costs being allocated to specialized addiction treatment services in Canada, and thus, will reduce the overall burden on Canadian society.

## Abbreviations

DATIS: Drug and alcohol treatment information system; FASD: Fetal alcohol spectrum disorder; FAS: Fetal alcohol syndrome

## Competing interests

The authors declare that they have no competing interests.

## Authors’ contributions

SP led the conception and design of the study, acquired the data, analyzed and interpreted the data, drafted and revised the manuscript, and has provided final approval of the version to be published. SL contributed to the conception and design of the study, contributed to the analysis and interpretation of the data, helped draft and revise the manuscript, and has provided final approval of the version to be published. LB assisted in the analysis and interpretation of the data, revised the manuscript critically for important intellectual content, and has provided final approval of the version to be published. KU collaborated on data analysis and interpretation, revised the manuscript critically for important intellectual content, and has provided final approval of the version to be published. JR was involved in the conception and design of the study, revised the manuscript critically for important intellectual content, and has provided final approval of the version to be published. All authors read and approved the final manuscript.

## Pre-publication history

The pre-publication history for this paper can be accessed here:

http://www.biomedcentral.com/1471-2458/13/570/prepub
